# The intellectual base and research fronts of IL-37: A bibliometric review of the literature from WoSCC

**DOI:** 10.3389/fimmu.2022.931783

**Published:** 2022-07-22

**Authors:** Ya-fei Qin, Shao-hua Ren, Bo Shao, Hong Qin, Hong-da Wang, Guang-ming Li, Yang-lin Zhu, Cheng-lu Sun, Chuan Li, Jing-yi Zhang, Hao Wang

**Affiliations:** ^1^ Department of General Surgery, Tianjin Medical University General Hospital, Tianjin, China; ^2^ Tianjin General Surgery Institute, Tianjin Medical University General Hospital, Tianjin, China

**Keywords:** IL-37, bibliometric analysis, visualization, CiteSpace, immunity

## Abstract

**Background:**

IL-37 is a recently identified cytokine with potent immunosuppressive functions. The research fronts of IL-37 are worth investigating, and there is no bibliometric analysis in this field. The purpose of this study is to construct the intellectual base and predict research hotspots of IL-37 research both quantitatively and qualitatively according to bibliometric analysis.

**Methods:**

The articles were downloaded from the Web of Science Core Collection (WoSCC) database from the inception of the database to 1 April 2022. CiteSpace 5.8.R3 (64-bit, Drexel University, Philadelphia, PA, USA) and Online Analysis Platform of Literature Metrology (https://bibliometric.com/) were used to perform bibliometric and knowledge-map analyses.

**Results:**

A total of 534 papers were included in 200 academic journals by 2,783 authors in 279 institutions from 50 countries/regions. The journal *Cytokine* published the most papers on IL-37, while *Nature Immunology* was the most co-cited journal. The publications belonged mainly to two categories of Immunology and Cell Biology. USA and China were the most productive countries. Meanwhile, the University of Colorado Denver in USA produced the highest number of publications followed by Radboud University Nijmegen in the Netherlands and Monash University in Australia. Charles A. Dinarello published the most papers, while Marcel F. Nold had the most co-citations. Top 10 co-citations on reviews, mechanisms, and diseases were regarded as the knowledge base. The keyword co-occurrence and co-citations of references revealed that the mechanisms and immune-related disorders were the main aspects of IL-37 research. Notably, the involvement of IL-37 in various disorders and the additional immunomodulatory mechanisms were two emerging hotspots in IL-37 research.

**Conclusions:**

The research on IL-37 was thoroughly reviewed using bibliometrics and knowledge-map analyses. The present study is a benefit for academics to master the dynamic evolution of IL-37 and point out the direction for future research.

## Introduction

IL-37, previously termed as IL-IF7, is a novel number of the IL-1 family discovered by computational prediction and renamed in 2010 (1). IL-37 is recently identified as a natural suppressor of innate immunity (2). The coding genes of IL-37, IL-1α, and IL-1β are also located on the long arm of chromosome 2 and are located closer following LPS stimulation (3). When pro-inflammatory signals induce the transcription of IL-1α and IL-1β genes, IL-37 transcription is also induced to limit excessive inflammation (3). The properties of this spatial structure are critical for the immunomodulatory effects of IL-37. Besides, it has been established that there is a “dual function” of IL-37 in that it is capable of acting intracellularly and extracellularly (4, 5). Above all, extracellular IL-37 bound to immobilized ligand-binding IL-18Rα and the decoy receptor IL-1R8 (5). Recombinant IL-37 resulted in a 16-fold increase in IL-1R8 mRNA levels in M1 macrophages. Beyond this, the expression level of TNF-α and IL-6 in mouse bone marrow-derived dendritic cells stimulated by LPS decreased by 50%–55% but not in an IL-1R8-deficient mice (5). The formation of the IL-37/IL-18Rα/IL-1R8 complex is a key mechanism by which IL-37 exerts its extracellular suppressive function (6). Notably, accumulating evidence indicated that endogenous IL-37 was effectively induced by inflammatory stimulation, which inhibited the production of IL-6, TNF-α, IL-1β, and protected mice against LPS-induced shock (7). Similar to other members of the IL-1 family, IL-37 has a caspase-1 cleavage domain at the aspartate site of the N-terminal sequence of exon 1 (8). The caspase-1 processes IL-37 precursors into mature IL-37 (9). The mature IL-37 then forms a compound with phosphorylated Smad3, which translocate into the nucleus exerting actual anti-inflammatory effects (10). It has been demonstrated that silencing Smad3 in IL-37-overexpressing RAW264.7 and THP-1 cell lines diminishes the anti-inflammatory role of IL-37, thereby inducing TNF-α and IL-6 expression (11). Similar findings have been validated in IL-37tg mice (11). Given broad immunomodulatory roles, IL-37 has been shown to be directly implicated in several inflammatory diseases, such as systemic lupus erythematosus and inflammatory bowel disease (12, 13). Due to its potent effect for inhibiting innate and adaptive immune responses, our recent publication revealed that IL-37 was also closely associated with cancer development such as oral squamous cell carcinoma, colorectal cancer, and hepatocellular carcinoma (14–16). With a constantly expanding number of articles, IL-37 has piqued the interest of academics. Many scholars have reviewed IL-37 research from various aspects. Su (17) summarized the expression and release of IL-37, IL-37-dependent intracellular and extracellular signaling, genetic variants, pathophysiological effects, IL-37-induced effects on cholesterol homeostasis, regulation of innate and acquired immunity, the role of IL-37 in mesenchymal stem cell, immune cells, autoimmune diseases, and cancer. Nevertheless, to the best of our knowledge, there are no reports with a comprehensive analysis of publication trends, authoritative author and institutions, and their cooperation, knowledge domain, and emerging trends in IL-37 research.

Bibliometrics refers to the quantitative analysis of scholar publications (18, 19). Bibliometrics could be used to qualitatively and quantitatively review relevant research from this period, comprehensively sort out the context of the IL-37 study, and encourage and offer new insights into IL-37 research (20). As far as we are aware, currently there is no bibliometric analysis of IL-37 research. This study aimed to use CiteSpace software and Online Analysis Platform of Literature Metrology to objectively describe the knowledge domain and emerging trends of IL-37 research. We quantified and identified the general information in IL-37 research such as annual publication trends, cooperation information, and the impact of author, institution, and country. Otherwise, we conducted the most co-cited references and keywords to assess the intellectual base of IL-37. Most of all, co-cited references, keyword bursts, and clustering were performed to detect the hotspots evolution and emerging topics.

## Materials and methods

### Data collection

Journal publications have strong timeliness and could accurately reflect the progression and changes of a research topic. The Web of Science Core Collection (WoSCC) bibliographic database is among the largest and most comprehensive electronic scientific literature databases worldwide (21). The retrieved documents in this database ensure the reliability and authority of the conclusions. The data were collected from the inception of the database to 1 April 2022 (22). The retrieval strategy used in this study was set to TS = (“IL-37” OR “IL-1F7” OR “interleukin 37” OR “interleukin 1 family, member 7 (zeta)” OR “IL1F7”). The article type was limited to Article or Review, and the language was confined to English. Then, the search results were recorded with the content of “Full Record and Cited References” in the format of “Plain Text.”

### Data analysis and visualization

We exported the citation reports and search results from the WoSCC database including the number of annual published articles, the annual citation number, the number of publications and H-indexes in different counties and institutions, and the quantity of categories. The downloaded files were imported into Microsoft Excel 2021 (version 16.48), CiteSpace 5.8.R3 (64-bit, Drexel University, Philadelphia, PA, USA), and Online Analysis Platform of Literature Metrology (https://bibliometric.com/) for further analysis (23, 24). CiteSpace, developed by Prof. Chao-mei Chen, has been the most applied bibliometrics software particularly adept at detecting the intellectual structure, research frontiers and dynamics, publication trend, and cooperation (25). Therefore, CiteSpace was performed to analyze and visualize co-cited references, co-cited authors, co-cited journals, cited reference bursts, co-occurrence of keywords, citation bursts for keywords, dual maps of journals, keyword clusters, co-cited reference clusters, and timelines. The specific settings used in CiteSpace were established as follows: time slicing was from January 2001 to December 2022 and years per slice = 1. Text processing, node types, link strength and scope, and selection criteria followed the default. Minimum spanning trees and pruning sliced networks were selected during the analysis process of keyword co-occurrence and burst. Pathfinder and pruning sliced networks were selected in the analysis of the co-cited reference cluster and other indicators. Microsoft Excel 2021 was used to visualize the annual publication number and citation number. Online Analysis Platform of Literature Metrology provided the bibliometric analysis of scientific citation data in an intuitive and friendly manner. The publication numbers among authors, institutions, journals, and collaboration between countries were visualized and analyzed based on this platform.

## Results

### Obtaining relevant literature

**Figure 1:**
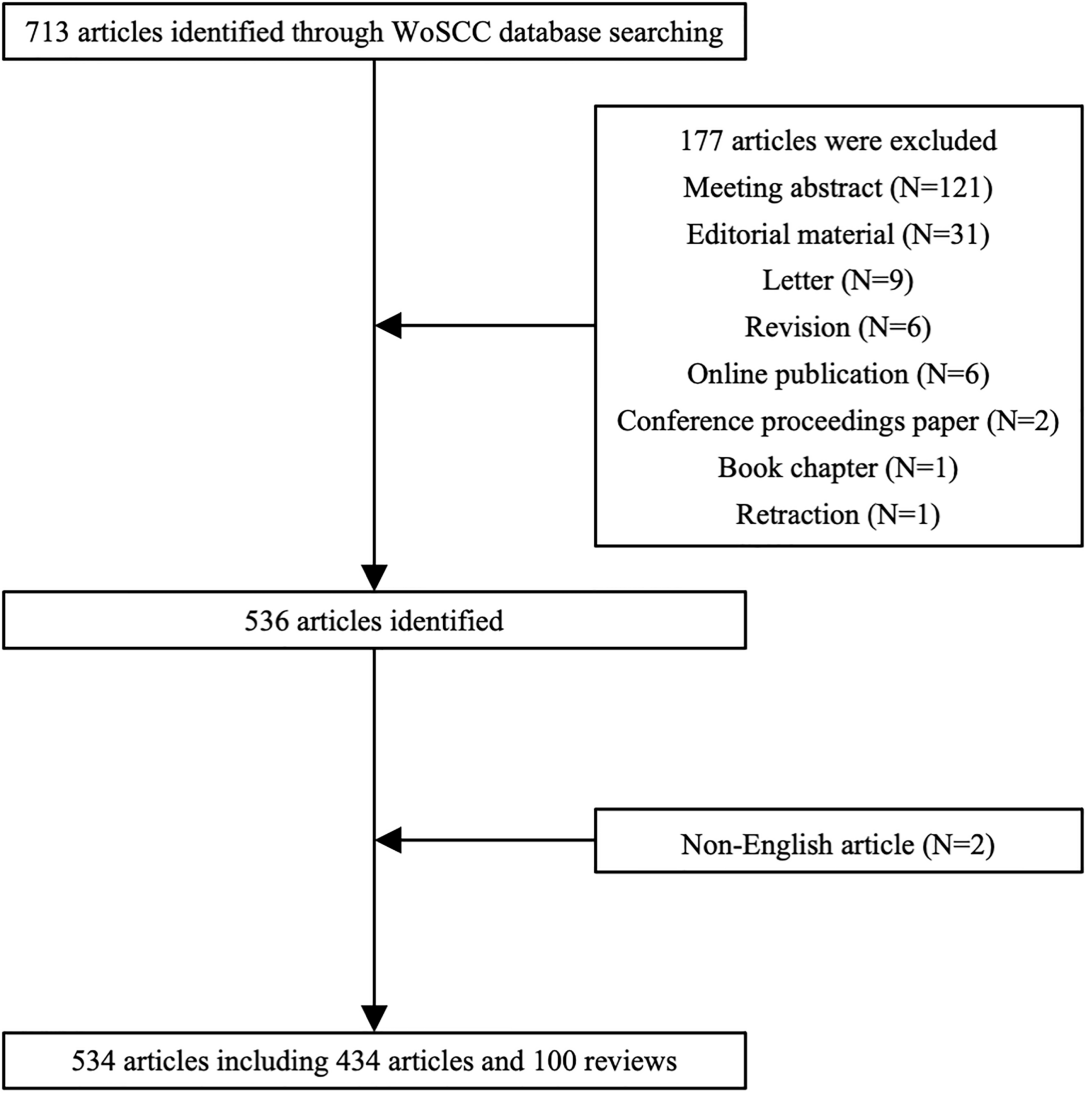
Flowchart of the screening process.

A total of 713 articles were identified through the WoSCC database initially. One hundred seventy-seven articles were ruled out based on the exclusion criteria including 121 meeting abstracts, 31 editorial materials, nine letters, six revisions, six online publications, two conference proceeding papers, one book chapter, one retraction, and two non-English articles. Five hundred thirty-four articles including 434 articles and 100 reviews were ultimately included in the present research (**Figure 1**).

### Annual publication growth trend

**Figure 2:**
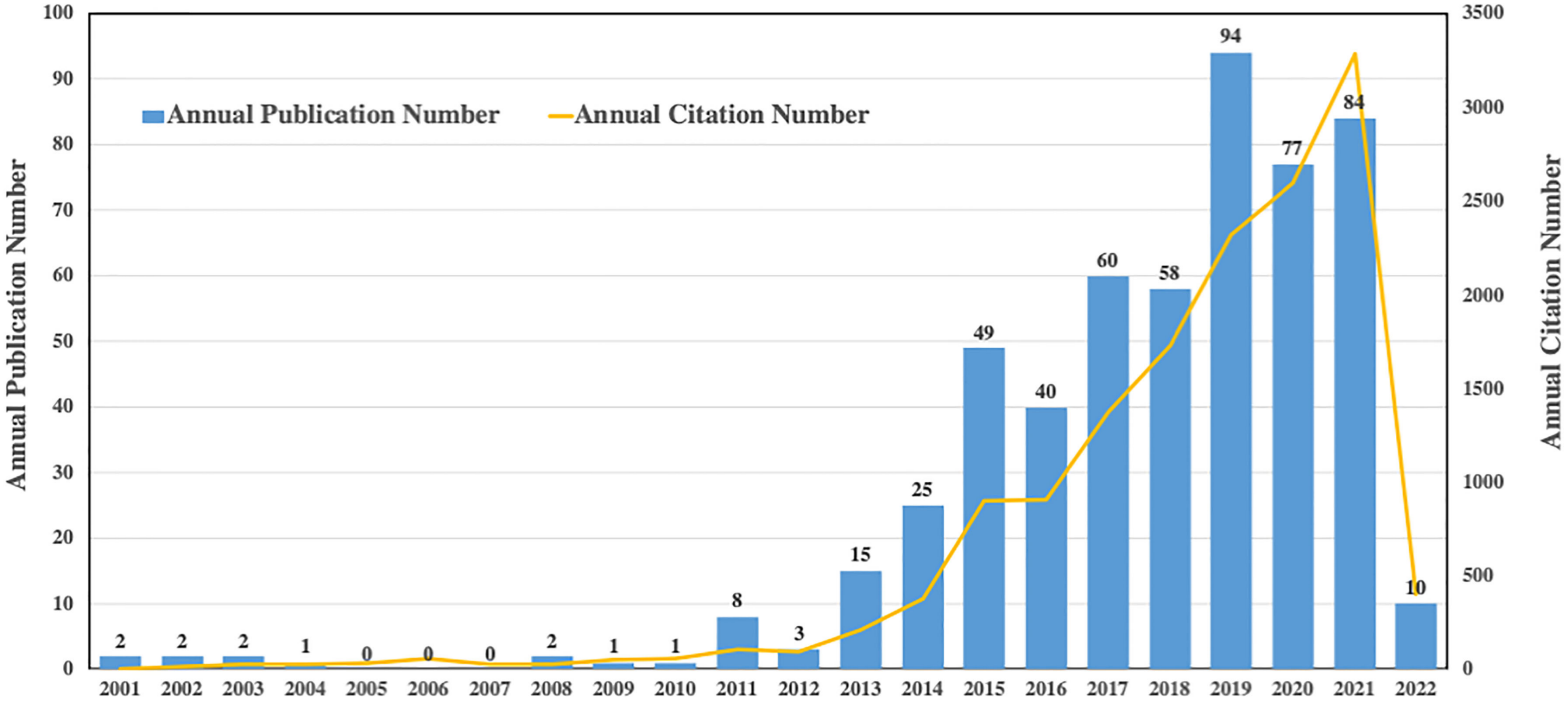
Annual publication growth trend of IL-37 research.

As we have known, IL-37 belongs to the IL-1 family and could also be referred to as IL-1F7 (26). The literature search yielded only 22 articles during the time period from 2001 to 2012 (**Figure 2**). Strikingly, the number of papers published on IL-37 topics overall continuously increased from 2013 to 2021 in a fluctuating manner, with a slight drop in 2016 and 2021. The highest annual number of published articles was more than 90. Phenomenally, the annual citation number has steadily increased from 2013 to 2021. Collectively, these results suggested that the research on IL-37 is appealing and that more prospective studies in the field of IL-37 are still needed in the future.

### Spatial distribution map of countries/regions, institutions, and collaboration

**Figure 3:**
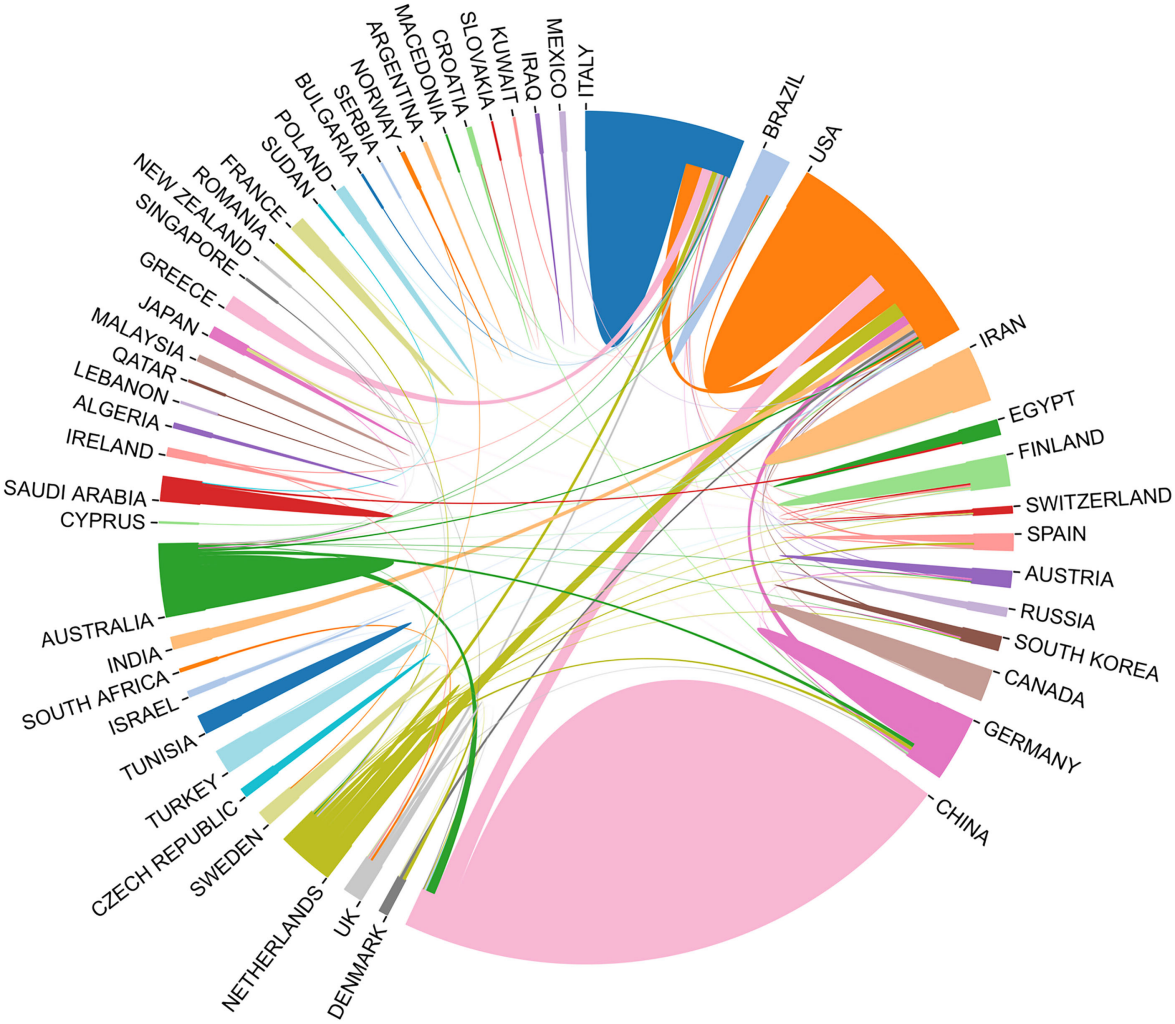
The network map of collaboration between countries/regions.

Fifty countries and 279 institutions have contributed research on the IL-37 field. As summarized in **Table 1**, the top 10 productive countries/regions and institutions were ranked. It is noteworthy that China, USA, Italy, Netherlands, and Germany are among the top 5 countries in the world in terms of IL-37 research, with China (257/48.1%) ranking first, followed by USA (23.2%) and Italy (10.5%). Although China has contributed nearly half the number of publications in the research of the IL-37 field, the total number of citations was 2,709, which is far less than that of USA (6455). The H-index was computed by taking the highest number of H such that H publications have at least H citations (27). Unsurprisingly, USA has the highest H index of 39, which is far higher than those of China (33) Italy (24), and Netherlands (23). The above data indicated that USA was the most influential country, far ahead of the rest in the research of the IL-37 field. The University of Colorado Denver (48, 9.0%) from USA produced the highest number of publications in IL-37 literature followed by Radboud University Nijmegen (47, 8.8%) in the Netherlands and Monash University (43, 8.1%). Besides, among the top 10 productive institutions, Fudan University, Shandong University, Huazhong University of Science and Technology, Guangdong Medical University, and Shenzhen University are all from China. It should also be noted that several affiliations such as the University of Colorado Denver (0.11), Radboud University Nijmegen (0.11), Huazhong University of Science and Technology (0.21), and Shenzhen University (0.21) showed high centrality, which means that these institutions occupy an important position in the research of the IL-37 field. As shown in **Figure 3**, collaboration network analysis revealed that the most frequent collaboration occurred between USA and China, followed by USA and the Netherlands.

**Table 1:** The top 10 countries/regions and institutions contributing to publications of IL-37 research.

Rank	Country	Number of publications	Number of citations	Citations of per article	H-Index	Institutions	Article counts	Total number of citations	Average number of citations	Total number of first authors	Total number of first author citations	Average number of first author citations	Location	Centrality
1	CHINA	257	2,709	16.22	33	Univ Colorado Denver	48	2,703	56.31	15	1,081	72.07	USA	0.11
2	USA	124	6,455	58.66	39	Radboud Univ Nijmegen	47	1,072	22.81	11	143	13	NETHERLANDS	0.11
3	ITALY	56	3,342	63.3	24	Monash Univ	43	2,150	50	5	39	7.8	AUSTRALIA	0.07
4	NETHERLANDS	46	3,261	75.35	23	Fudan Univ	39	255	6.54	14	91	6.5	CHINA	0.08
5	GERMANY	36	2,607	77.06	21	Shandong Univ	38	365	9.61	16	181	11.31	CHINA	0.04
6	AUSTRALIA	26	1,764	71.04	15	Huazhong Univ Sci & Technol	31	394	12.71	14	190	13.57	CHINA	0.20
7	ENGLAND	18	1,213	67.56	12	Tufts Univ	28	99	3.54	5	18	3.6	USA	0.01
8	GREECE	17	182	12.24	10	Univ G dAnnunzio	28	91	3.25	17	58	3.41	SOUTH KOREA	0.03
9	IRAN	16	135	8.75	8	Guangdong Med Univ	27	90	3.33	10	47	4.7	CHINA	0.01
10	SOUTH KOREA	15	847	58.4	10	Shenzhen Univ	25	780	31.2	10	272	27.2	CHINA	0.21

### Visual analysis of journals and co-cited journals

Online Analysis Platform of Literature Metrology was applied to identify the most productive and influential journals in the field of IL-37 research. The results show that 534 articles were published in 200 academic journals. As shown in **Table 2**, *cytokine* (3.861, Q3) published the most papers (26, 4.9%) with 231 total citations, followed by *Frontiers in Immunology* (7.561, Q1), *Scientific Reports* (4.38, Q1), and *Mediators of Inflammation* (4.711, Q2). Although only 13 articles were published in *Proceedings of the National Academy of Sciences of the United States of America* (PNAS, 11.205, Q1), it received 726 citations which are threefold more than the journal of *Cytokine*. Among the top 10 journals, four were at the Q1 JCR division and four had an impact factor (IF) of more than 5. Among 519 co-cited journals, seven journals had citations over 200. *Nature Immunology* (25.606, Q1) had the most co-citations, followed by *Journal of Immunology* (5.422, Q2), *PNAS* (11.205, Q1), and *Cytokine* (3.861, Q3). Among the top 10 co-cited journals, four were at the Q1 JCR division, and five had an IF of more than 5.

**Table 2:** The top 10 most productive journals and co-cited journals.

Rank	Journals	Counts	Total number of citations	Average number of citations	IF and JCR division (2020)	Co-cited journals	Total number of co-citations	IF and JCR division (2020)	Centrality
1	Cytokine	26	231	8.88	3.861, Q3	Nature Immunology	427	25.606, Q1	0.01
2	Frontiers in Immunology	21	70	3.33	7.561, Q1	Journal of Immunology	383	5.422, Q2	0.01
3	Scientific Reports	18	191	10.61	4.38, Q1	PNAS	383	11.205, Q1	0.02
4	Mediators of Inflammation	14	120	8.57	4.711, Q2	Cytokine	279	3.861, Q3	0.01
5	PNAS	13	726	55.85	11.205, Q1	Plos One	256	3.24, Q2	0.00
6	International Immunopharmacology	13	69	5.31	4.932, Q2	Journal of Biological Chemistry	222	5.157, Q2	0.01
7	International Journal of Molecular Science	10	29	2.9	5.924, Q1	European Cytokine Network	216	2.737, Q3	0.01
8	Journal of Immunology	7	351	50.14	5.422, Q2	Immunity	199	31.745, Q1	0.02
9	Plos One	7	94	13.43	3.24, Q2	SCI REP-UK	190	4.38, Q1	0.01
10	Journal of Interferon and Cytokine Research	7	68	9.71	2.607, Q3	Clinical and Experimental Immunology	186	4.33, Q2	0.03

**Figure 4:**
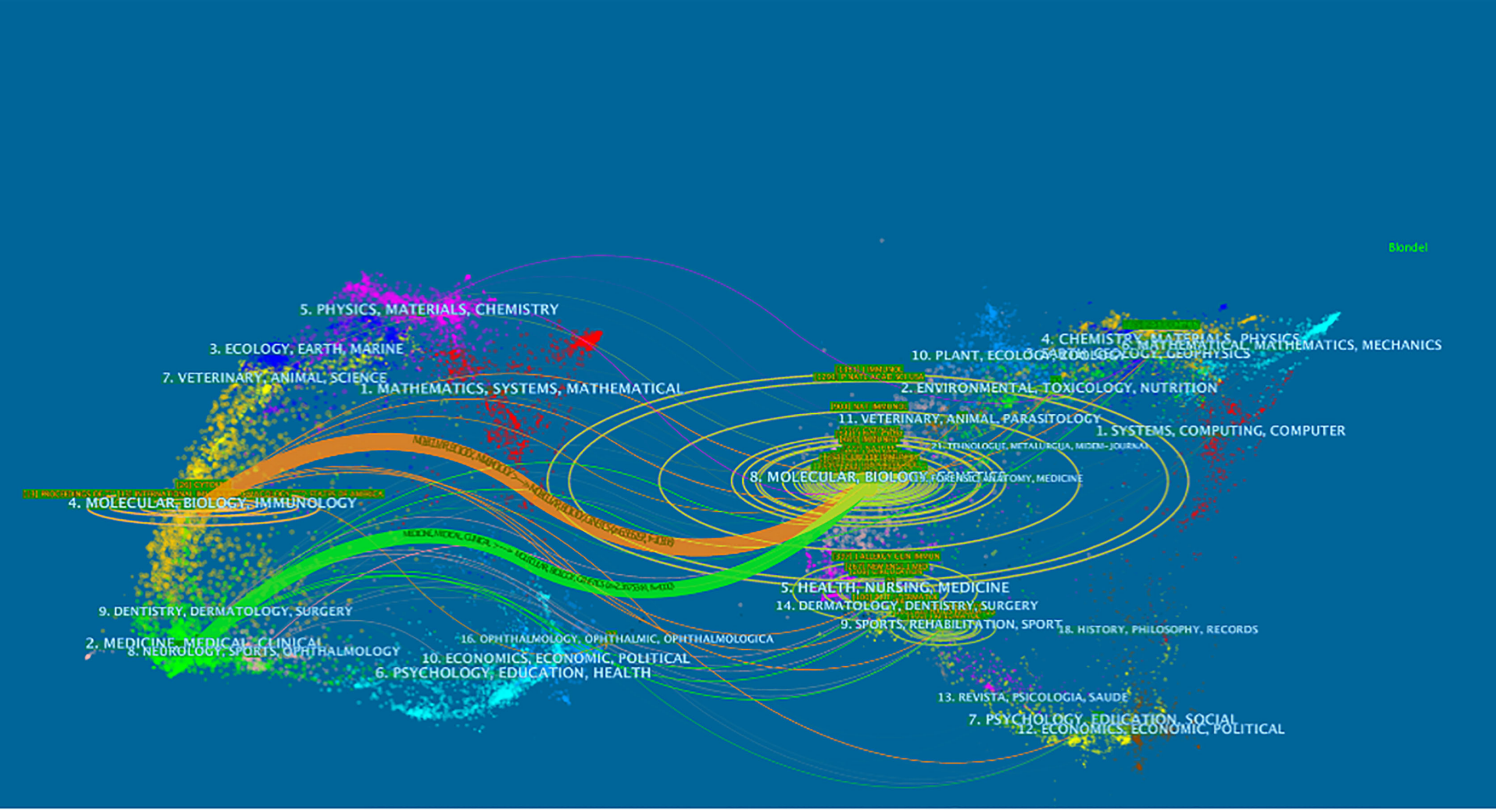
The dual-map overlay of journals.

Knowledge flow analysis is used to explore the evolutionary relationship of knowledge citations and co-citations between citing and cited journals (28). The dual-map overlay of journals can intuitively show the distribution of journals in various disciplines, the evolution of citation trajectories, and the drift of scientific research centers (28, 29). It is a new method to show the flow of knowledge at the journal level on the dual-map overlay of journals. The citing map is on the left and the cited map is on the right. All colored curves originating from the citing map and pointing to the ones cited are the citation connection lines, which completely show the ins and outs of the citation. In the citing map, the more papers the journal publishes, the longer the vertical axis of the ellipse, and the greater the number of authors, the longer the horizontal axis of the ellipse (28). **Figure 4** in the present research indicates that the articles published in Molecular/Biology/Genetics journals are often cited by Medicine/Medical/Clinical journals and Molecular/Biology/Immunology journals.

### Analysis of authors and co-cited authors

Co-cited author analysis refers to when the literatures of two authors are simultaneously cited by a third author. The higher the number of co-citations, the closer their academic interests are, and the closer the research density is (30). Analysis of the most productive author and co-cited author in the IL-37 field research could intuitively reflect the research strength and hot topics of research. The 534 selected publications were produced by 2,783 authors, and the top 10 productive authors and co-cited authors are listed in **Table 3**. Charles A. Dinarello (Department of Medicine, Radboud University Medical Center and Department of Medicine, University of Colorado Denver) headed with 46 documents and 2,218 total citations, followed by Conti with 24 documents with a total of only 79 of citations. Bufler from the Department of Medicine, University of Colorado Denver ranked third in productive author with 20 documents and 1,718 total citations. We also note that although only 15 articles have been published by Marcel F. Nold (Department of Medicine, University of Colorado Denver), 1,283 citations were cited. As we have seen, Charles A. Dinarello, Bufler, and Marcel F. Nold, productive authors with the most total number of citations, are all from the University of Colorado Denver, implying that the University of Colorado Denver produced chief articles in this field. Meanwhile, we cannot ignore that five out of 10 authors have centrality less than 0.01, which means that these authors should strengthen cooperation with others. It is demonstrated in **Table 3** that the most frequently co-cited author is Marcel F. Nold (356 citations), who is also the seventh most productive, right after whom Charles A. Dinarello appears with 211 citations, which is also the most productive author. The following authors are Boraschi (203 citations), McNamee (179 citations), and Claudia A. Nold-Petry (154 citations). Indeed, China has produced the largest number of literatures in this field. However, the most productive and co-cited authors are American scholars. It could be that numerous fundamental studies have been carried out by American scholars particularly at an early stage.

**Table 3:** The top 10 most productive authors and co-cited authors contributed to IL-37 research.

Rank	Author	Article counts	Total number of citations	Average number of citations	First author counts	First author citation counts	Average first author citation counts	Corresponding author	Corresponding author citation counts	Centrality	Co-cited author	Citation counts	Centrality
1	Dinarello, CA	46	2,218	48.22	3	178	59.33	13	1,180	0.06	Nold MF	356	0.01
2	Conti, P	24	79	3.29	18	58	3.22	18	58	0.00	Dinarello CA	211	0.12
3	Bufler, P	20	1,718	85.9	1	100	100	6	302	0.01	Boraschi D	203	0.01
4	Ronconi, G	19	58	3.05	0	0	0	0	0	0.01	McNamee EN	179	0.02
5	Caraffa, A	18	58	3.22	0	0	0	0	0	0.00	Nold-Petry CA	154	0.00
6	Kritas, SK	16	50	3.13	0	0	0	0	0	0.00	Kumar S	154	0.04
7	Nold, MF	15	1,283	85.53	1	360	360	4	190	0.01	Bufler P	151	0.13
8	Garlanda, C	14	518	37	1	64	64	1	0	0.02	Ye L	145	0.00
9	Theoharides, TC	14	59	4.21	2	8	4	5	17	0.00	Bulau AM	142	0.08
10	Huang, Z	13	294	22.62	2	29	14.5	4	105	0.00	Li SZ	122	0.02

### Co-cited reference analysis and clustering network

**Figure 5:**
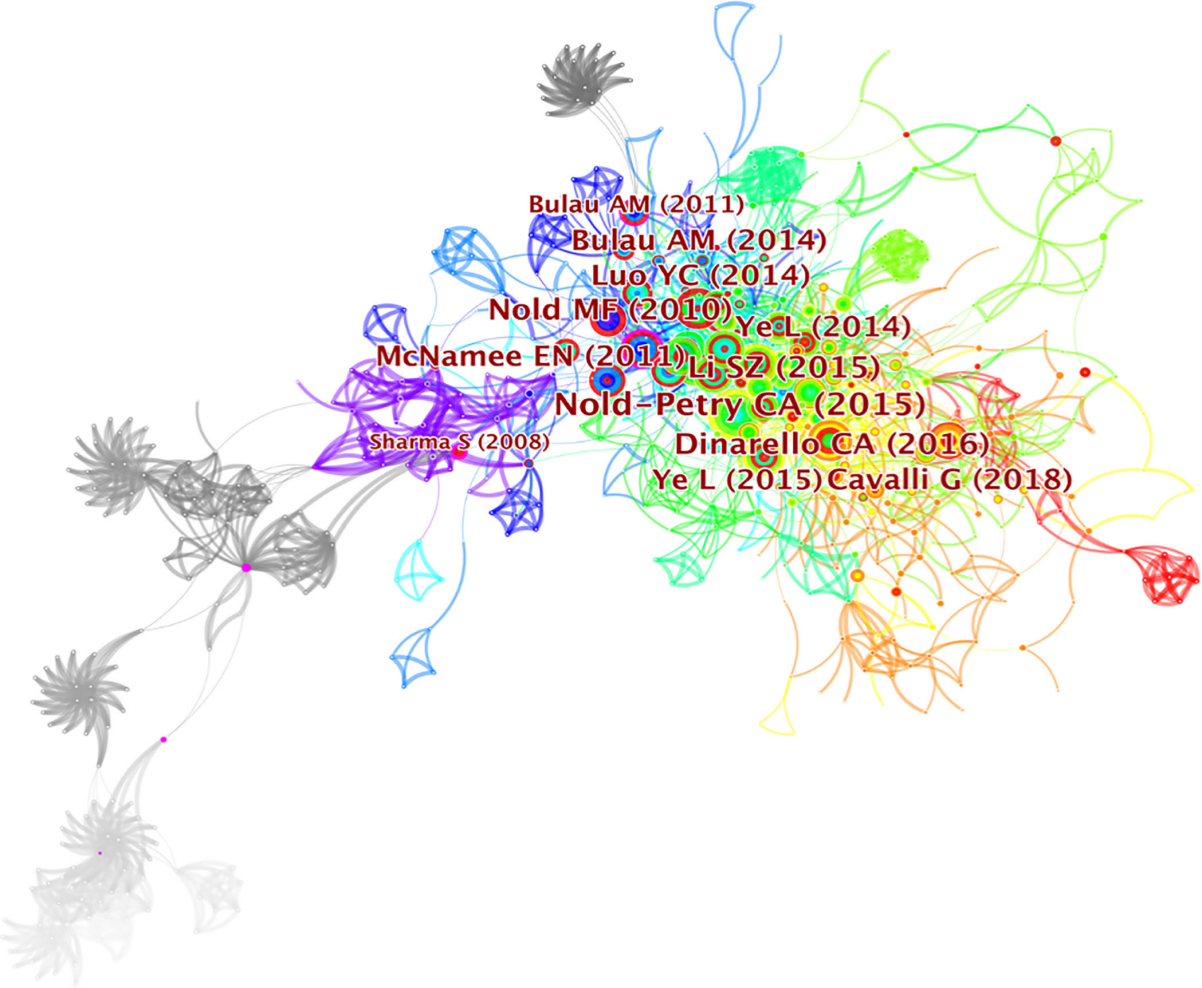
The visualization of co-cited references on IL-37 research.

Literature co-citation analysis expresses the relationship between documents by analyzing the frequency at which they are cited (31). The knowledge base consists of a collection of co-cited articles, while research fronts consist of collections of citing articles (32). The clusters of co-cited references in CiteSpace were determined by extracting titles of citing articles using a log‐likelihood ratio algorithm (33). The clusters of co-cited references were deemed as research fronts (34). A total of 776 co-cited references were visualized by CiteSpace and are demonstrated in **Figure 5**. The first author and the top 12 most cited references are clearly seen on the graph. The size of circles is proportional to the co-citation frequency. The thickness of a ring of circles is directly proportional to the number of co-citations in the corresponding time zone. The thicker the ring, the more co-citations in the time zone. The color of the link between the two circles represents the year of the first co-citation for two references. **Table 4** presents the details of top 10 co-cited references in the field of IL-37 research. The most co-cited reference performed by Claudia A. Nold-Petry et al. (35) in 2015 was an original article published in *Nature Immunology*, entitled “IL-37 requires the receptors IL-18Rα and IL-1R8 (SIGIRR) to carry out its multifaceted anti-inflammatory program upon innate signal transduction”, followed by an article entitled “Extracellular forms of IL-37 inhibit innate inflammation *in vitro* and *in vivo* but require the IL-1 family decoy receptor IL-1R8” (5). The two articles respectively discuss the extracellular mechanism of IL-37 and assessed its role in innate immunity. The results of two studies set the basis for further research on IL-37. The five frequently cited literatures are from USA, and the first five are from the University of Colorado Denver. Therefore, the University of Colorado Denver attached importance of IL-37 research not only to the quantity but also to the quality of the articles. In contrast, it is not ignored that the nine out of top 10 co-cited references with centrality less than 0.1, suggesting that the scholars on IL-37 research lack cooperation and academic exchanges. The co-cited reference entitled “Interleukin 37 expression protects mice from colitis” with high betweenness centrality (0.21) could be identified as a vital point that provides important bridging connections between two research interests.

**Table 4:** The top 10 co-cited references of IL-37 research.

Rank	Title	Year	Author	Number of co-citations	Centrality
1	IL-37 requires the receptors IL-18Rα and IL-1R8 (SIGIRR) to carry out its multifaceted anti-inflammatory program upon innate signal transduction	2015	Claudia A Nold-Petry	117	0.01
2	Extracellular forms of IL-37 inhibit innate inflammation *in vitro* and *in vivo* but require the IL-1 family decoy receptor IL-1R8	2015	Suzhao Li	98	0.08
3	Suppression of innate inflammation and immunity by interleukin-37	2016	Charles A. Dinarello	84	0.01
4	IL-37 is a fundamental inhibitor of innate immunity	2010	Marcel F Nold	83	0.09
5	Suppression of inflammation and acquired immunity by IL-37	2017	Giulio Cavalli	77	0.06
6	Suppression of antigen-specific adaptive immunity by IL-37 *via* induction of tolerogenic dendritic cells	2014	Yuchun Luo	75	0.06
7	Role of caspase-1 in nuclear translocation of IL-37, release of the cytokine, and IL-37 inhibition of innate immune responses	2014	Ana-Maria Bulau	74	0.09
8	IL-37 inhibits the production of inflammatory cytokines in peripheral blood mononuclear cells of patients with systemic lupus erythematosus: its correlation with disease activity	2014	Liang Ye	74	0.00
9	Interleukin 37 expression protects mice from colitis	2011	Eóin N. McNamee	73	0.21
10	IL-37 Alleviates Rheumatoid Arthritis by Suppressing IL-17 and IL-17-Triggering Cytokine Production and Limiting Th17 Cell Proliferation	2015	Liang Ye	70	0.04

**Figure 6:**
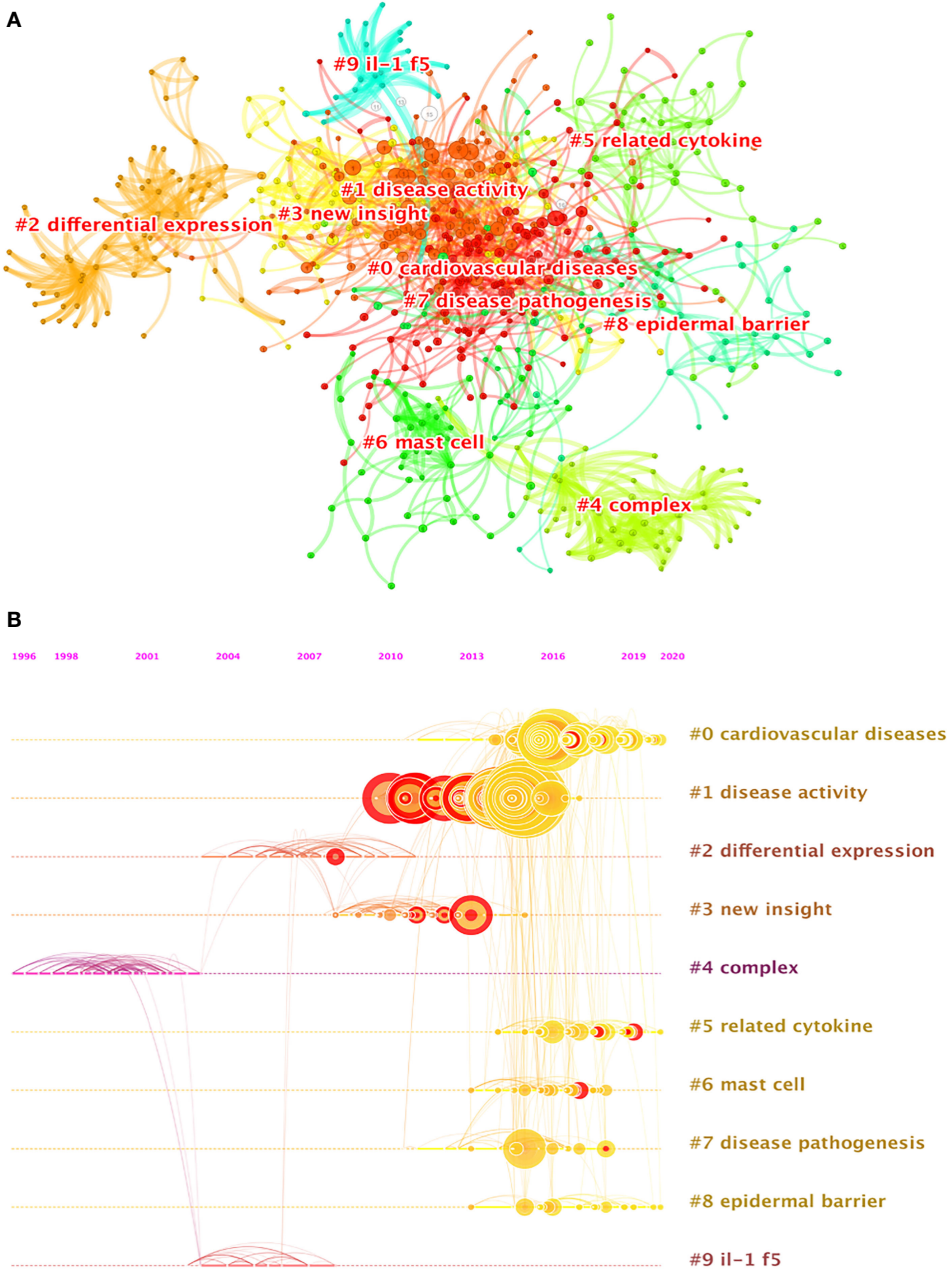
**(A)** The clustered network map of co-cited references on IL-37 research. **(B)** The timeline view of co-citation clusters.

The cluster analysis of literature co-citation could objectively show the knowledge structure of the research field. On this basis, we further constructed a network map to visualize the top 10 clusters of co-cited references (**Figure 6A**). Pathfinder and pruning sliced networks were selected to remain the most salient network structure when the co-cited references were clustered. Modularity was 0.799, and the weighted mean silhouette was 0.9156. Therefore, the results of the cluster in the present research are compelling. Clearly, all the clusters shown in **Table 5** are highly homogeneous with silhouette values above 0.7, suggesting a reliable clustering result. Cluster #0, namely, cardiovascular disease, was the largest cluster consisting of 123 references, followed by disease activity (cluster #1), differential expression (cluster #2), new insight (cluster #3), complex (cluster #4) and related cytokine (cluster #5), mast cell (cluster #6), disease pathogenesis (cluster #7), epidermal barrier (cluster #8), and IL-1f5 (cluster #9). To reveal the research trends and hotspots over time, we conducted a timeline view of co-cited references (**Figure 6B**). It is obvious that clusters#1 have a high concentration of nodes with citation bursts from 2010. Cluster #0 has a sustained period of about 10 years from 2010 to 2020, whereas cluster #2 is short-lived with an associated period of 7 years from 2008 to 2015. The results exhibited that cluster #1 has the largest nodes scattered along the timeline and contains 8 of the 10 most frequently cited references (2, 4, 5, 12, 35–38). Besides, it is obvious that the research on IL-1F5 (SIGIRR, cluster #9) with the size of 21 publications started earlier and relevant studies has a certain interruption period. Furthermore, cluster #4 and cluster#9 are separate from other clusters with infrequent citation links connecting to other clusters.

**Table 5:** Temporal major clusters of co‐cited references.

Cluster ID	Size	Silhouette	Mean (year)	Label (LSI)	Label (LLR)	Label (MI)
0	124	0.784	2017	cardiovascular diseases; human health	cardiovascular diseases; human health	IL-1f5; IFN-γ
1	115	0.864	2013	disease activity; potential role	disease activity; mast cell	IL-1f5; IFN-γ
2	62	0.994	2007	differential expression	differential expression	IL-1f5; IFN-γ
3	60	0.922	2010	new insight; IL-1 family member	new insight; interleukin-1 family	IL-1f5; IFN-γ
4	49	0.992	2000	IL-18 activity; complex	complex; IL-18 activity	disease activity; potential role
5	47	0.932	2017	IL-1 family cytokine; related cytokine	related cytokine; IL-1 family cytokine	IL-1f5; IFN-γ
6	44	0.974	2016	mast cell; inflammatory response	mast cell; key suppressor	allograft rejection; IL-1f5
7	34	0.913	2014	disease pathogenesis; human aortic valve	disease pathogenesis; human aortic valve	cervical cancer; cell apoptosis
8	32	0.969	2017	IL-1 family; IL-1 family cytokine	epidermal barrier; therapeutic perspective	IL-1 inhibitor
9	21	0.972	2004	IL-1f5 mediates anti-inflammatory activity	IL-1f5; induction	disease activity; potential role

### Burst detection of co-cited references

**Figure 7:**
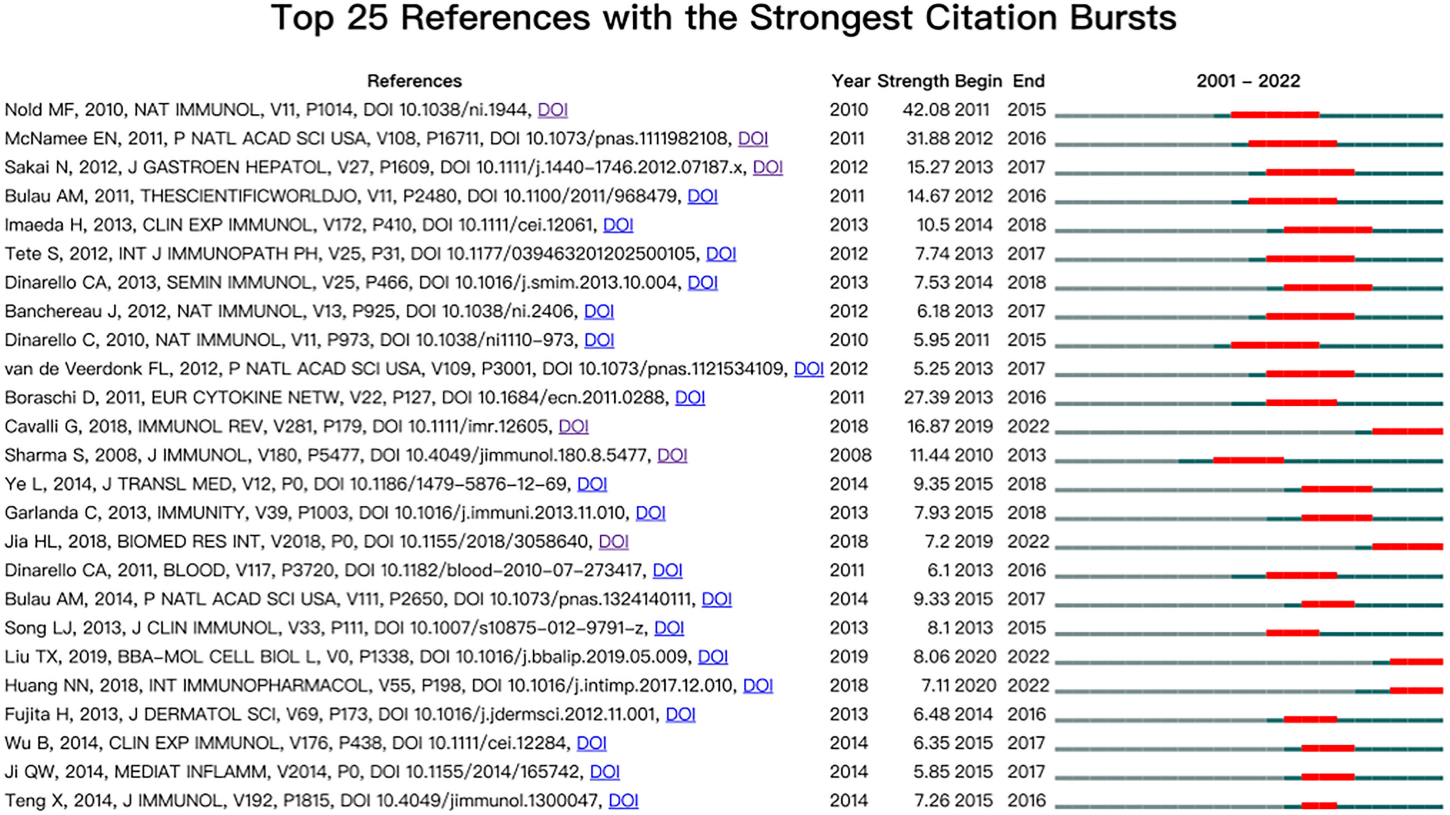
Top 25 references with the strongest citation bursts.

Co-cited reference bursts provide helpful insight into the research footprint of the research focus (39, 40). In CiteSpace, the number of states was stetted to 2, γ [0,1] = 1.0, and minimum duration = 1. The top 25 co-cited references with strong bursts were recorded (**Figure 7**). The first co-citation burst began in 2001, entitled “The IL-1 Family Member 7b Translocates to the Nucleus and Down-Regulates Proinflammatory Cytokines” (9). The paper with the strongest burst (strength = 42.08) was entitled “IL-37 is a fundamental inhibitor of innate immunity”, published in *Nature Immunology* by Marcel F. Nold in 2010, with bursts from 2011 to 2015 (2). The result showed that 34.55% (19/25) of the references showed citation burstiness in 2014, followed by 2013 (2/25, 27.8%) and 2015 (5/25, 24.0%). Remarkably, four references were in burstiness until 2022 which indicated that research related to IL-37 may continue to be explored in the future.

### Keyword co-occurrence and burst

**Figure 8:**
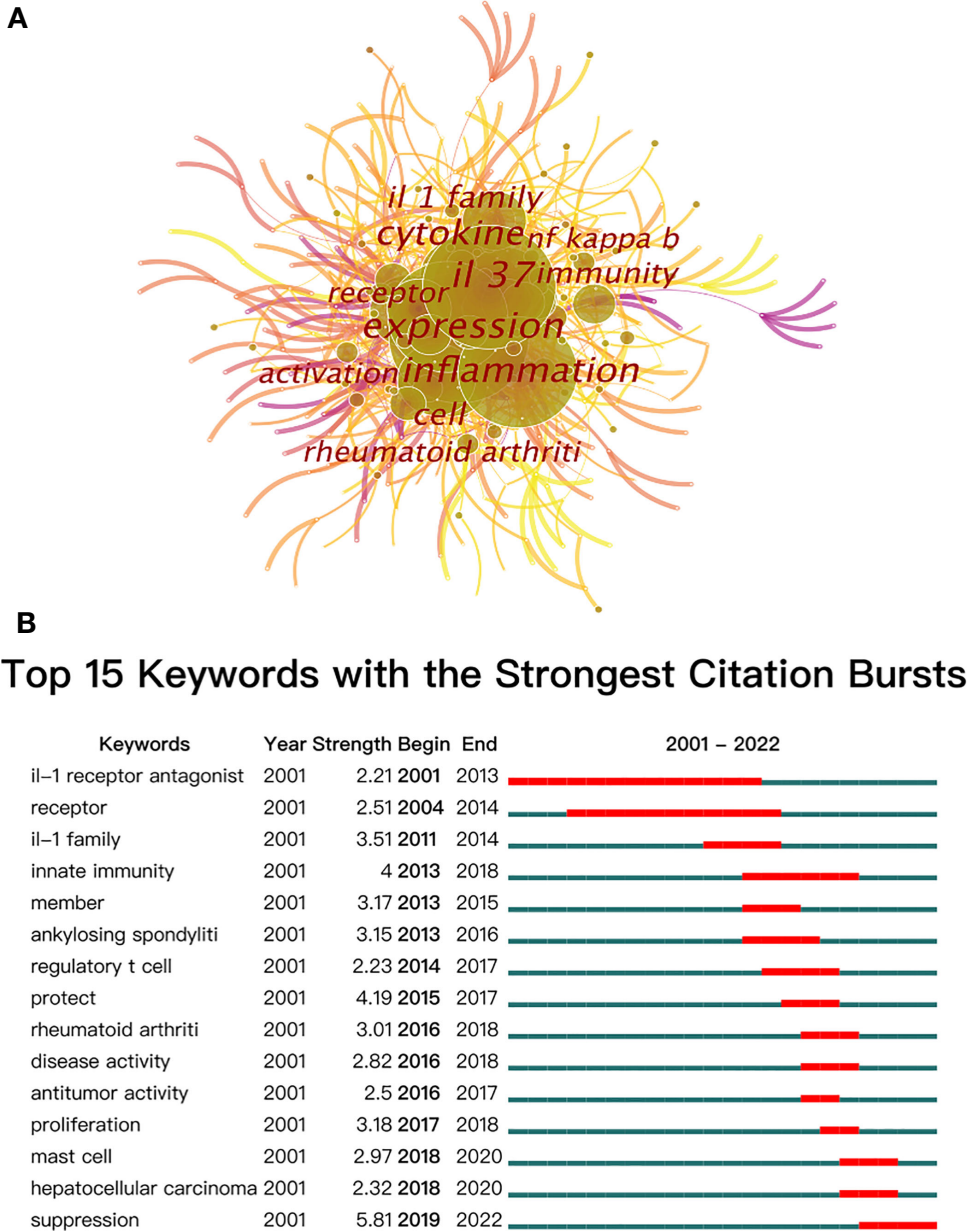
**(A)** The co-occurrence map of keywords on IL-37 research. **(B)** Top 15 keywords with the strongest citation bursts.

In bibliometrics, keyword co-occurrence and burst (**Figures 8A, B**) can reflect the emerging concepts that increased abruptly over time. A total of 423 keywords were extracted, of which 57 keywords appeared more than 10 times. As demonstrated in **Figure 8A** and **Table 6**, “IL-37” and “expression” were the most important terms with 165 co-occurrences, followed by “inflammation”, “cytokine”, “IL-1 family”, “cell”, and “immunity”. In addition, the centrality of “IL-37”, “inflammation”, “IL-1 family”, “receptor”, “NF-κB”, “pathogenesis”, “pathogenesis”, and “inhibitor” was greater than or equal to 0.1. Also, the burst for keywords revealed that “Suppression” is still the research front of IL-37. Considering the immunomodulatory role of IL-37, the function of IL-37 in inhibiting the potentiating immune responses in different disorders should be verified more in the future.

**Table 6:** The top 20 keywords of IL-37 research.

Rank	Keywords	Counts	Centrality	Year	Rank	Keywords	Counts	Centrality	Year
1	Expression	165	0.02	2002	11	NF-κb	46	0.11	2002
2	IL-37	165	0.14	2013	12	Disease	40	0.09	2014
3	Inflammation	134	0.15	2011	13	Pathogenesis	36	0.10	2013
4	Cytokine	117	0.07	2004	14	T cell	32	0.07	2004
5	IL-1 family	69	0.21	2003	15	Member	31	0.08	2002
6	Cell	66	0.08	2002	16	Innate inflammation	31	0.05	2017
7	Immunity	58	0.08	2011	17	Inhibitor	29	0.10	2001
8	Receptor	54	0.10	2004	18	IL-18Rα	29	0.05	2016
9	Rheumatoid arthritis	54	0.06	2013	19	Dendritic cell	27	0.14	2012
10	Activation	52	0.08	2002	20	Suppression	27	0.02	2018

### Subject categories

According to the results of WOS, the categories of Immunity and Cell Biology account for almost 50% of IL-37 research followed by Biochemistry Molecular Biology (16.854%), Medicine Research Experimental (9.738%), and Multidisciplinary Sciences (8.24%). The immunomodulatory effect of IL-37 and its role in different cells remain a major focus of IL-37 research (**Table 7**).

**Table 7:** Top 20 subject categories of IL-37 research.

Rank	Web of Science categories	Quantity	Percentage
1	Immunology	200	37.453
2	Cell Biology	108	20.225
3	Biochemistry Molecular Biology	90	16.854
4	Medicine Research Experimental	52	9.738
5	Multidisciplinary Sciences	44	8.24
6	Pharmacology Pharmacy	39	7.303
7	Oncology	37	6.929
8	Rheumatology	22	4.12
9	Allergy	19	3.558
10	Medicine General Internal	15	2.809
11	Chemistry Multidisciplinary	13	2.434
12	Endocrinology Metabolism	13	2.434
13	Dermatology	12	2.247
14	Biotechnology Applied Microbiology	11	2.06
15	Microbiology	11	2.06
16	Pathology	10	1.873
17	Genetics Heredity	9	1.685
18	Cardiac Cardiovascular Systems	8	1.498
19	Gastroenterology Hepatology	8	1.498
20	Hematology	8	1.498

## Discussion

To our knowledge, this would be the first bibliometric analysis of the intellectual base and research fronts of IL-37. As of 1 April 2022, 534 records were identified in 200 journals by 2,783 authors in 279 institutions from 50 countries/regions. The annual number of publications is an important indicator to measure the strength and development trend of IL-37 over a specific period (41, 42). An increasing trend of IL-37 research officially started in 2001. IL-37, previously termed IL-1F7, was discovered by computational cloning in 2000 (43). Before 2013, the number of studies investigating IL-37 function was relatively few. After that, the publications continued to rise in IL-37 research, which implies that IL-37 has also gained much attention. Increased levels of circulating IL-37 have been reported in the patients with rheumatoid arthritis (37, 44–48), multiple sclerosis (49–51), and systemic lupus erythematosus (12, 52–54). Patients with rheumatoid arthritis demonstrated an increased level of synovial IL-1R8, which is essential for the anti-inflammatory effects of IL-37. Not only that, it has been proven that IL-37 induces apoptosis in rheumatoid arthritis fibroblast-like synoviocytes (17, 55). Multiple sclerosis is a central nervous system-demyelinating illness that primarily affects young people (56). By acting through IL-18Rα and IL-1R8, neurological deficits and myelin loss in the experimental autoimmune encephalomyelitis model, an extensively used model for multiple sclerosis, were protected by the treatment of rhIL-37 (57). The MSCs overexpressing IL-37 decreased the levels of IL-1, TNF-α, IL-17, IL-6, anti-dsDNA, and anti-ANA in systemic lupus erythematosus mice (58). On the contrary, the abundance of circulating IL-37 decreased in patients with inflammatory bowel disease compared to healthy subjects (13). Likely, IL-37b gene transfer enhances the therapeutic efficacy of MSCs in DSS-induced colitis mice (59). In addition to the disorders described above, an increasing number of studies were performed to develop a better understanding regarding the role of IL-37 in other diseases including necrotizing enterocolitis (60), temporomandibular joint inflammation (61, 62), Behcet’s disease (63, 64), periodontitis (65), idiopathic pulmonary fibrosis (66), type 2 diabetes mellitus (67, 68), calcific aortic valve disease (69, 70), asthma (71–73), liver inflammation, and fibrosis (74). With increased in-depth IL-37 research, the role of IL-37 in different cells and relevant cell signaling pathways captured attention. Macrophage-orchestrated chronic inflammation plays a vital role in accelerating the development of calcific aortic valve disease. It has been proven that rhIL-37 shifts macrophage polarization from the pro-inflammatory M1 phenotype to the anti-inflammatory M2 phenotype by inhibiting the Notch1 and NF-kB pathways to suppress the progression of calcific aortic valve disease (75). *In vitro*, DCs from IL-37tg mice with downregulation of MHC II and CD40 molecular have diminished the ability to stimulate naïve T cells and activate antigen-specific T cells (36). Anti-inflammatory actions of IL-37 depend on inhibition of various pro-inflammatory actions and activation of anti-inflammatory signaling mediators. It has been proven that IL-37 binding to IL-1R8 inhibits the pro-inflammatory properties of the signaling molecules, including FAK, STAT1, p53, p38, paxillin, Pyk2, Syk, NLRP3, and SHP-2. Furthermore, PTEN is upregulated by IL-37 and thus may inhibit PI3K-AKT-mTOR, MAPK, and FADE pathways (2, 35, 76).

As shown in **Table 2**, the journals of *Cytokine* and *Nature Immunology* published the most papers about IL-37 research and received the largest number of co-citations, respectively. Most of the studies related to IL-37 research belong to the subject of biology and immunology, which is consistent with the dual-map analysis (**Figure 4**). Given the broad regulatory roles of IL-37 in innate and adaptive immunity, some journals concentrating on immunology research are enthusiastic about IL-37 research. It is worth noting that although *Cytokine* published the most IL-37 research, it took the fourth place in the journals ranking with the total number of co-citations.

These three journals, namely, *Nature Immunology*, *Journal of Immunology*, and *PNAS*, dominate the number of citations in the field with approximately 50% of all the co-citations. This is because fundamental IL-37 research has been published in these journals and gained tremendous attention. The spatial distribution map of institutions revealed that the University of Colorado Denver located in the USA is the most productive institution with 48 publications, 2,703 total citations, and high centrality, which plays a key bridge role in institution cooperation networks. Of note, the five most productive institutions Fudan University, Shandong University, Huazhong University of Science and Technology, Guangdong Medical University, and Shenzhen University are from China, of which Shenzhen University and Huazhong University of Science and Technology had high centrality. Although the five institutions are from China, interagency cooperation among them seems to be lacking. Moreover, we found active collaboration among the University of Colorado Denver and Radboud University Nijmegen, implying their significant contributions to the IL-37 field. A comprehensive look at the above results in **Table 1** demonstrates that USA and China are productive and influential countries in IL-37 research. Furthermore, USA was the earliest country to conduct the IL-37 study, followed by Germany, Italy, South Korea, and Australia, the top 10 productive countries. Although China started later than in the above four countries, it has emerged as one of the most productive contributors. This also provides a potential interpretation for the greatest number of publications, almost twice that of USA.

Detecting the top co-occurrences and co-cited papers in the specific field could benefit the research guideline and further directions (77). Three writers were co-cited more than 200 times among the total co-cited authors, with Marcel F. Nold receiving the most co-citations (356 citations), followed by Charles A. Dinarello (211 citations) and Boraschi (203 citations). In addition, Marcel F. Nold, Bufler, and Charles A. Dinarello were not only the top 10 productive authors but also the top 10 co-cited authors, indicating that their work contributed immensely to understanding the role of IL-37. In particular, Marcel F. Nold ranks seventh in the co-occurrence of authors but with the most co-citations. A vital paper entitled “IL-37 is a fundamental inhibitor of innate immunity” published by Marcel F. Nold et al. (2) in 2010 was constructive and profoundly significant to the IL-37 research. It has to be mentioned that although Chinese academics contribute a large quantity of publications of IL-37, the productive authors are especially scarce. The more meticulous research on IL-37 would be the need for Chinese scholars.

Co-cited references appear together in the bibliography of a third citing paper, and the two papers constitute a co-citation relationship (29, 78). The intellectual base and research fronts are the integration of co-cited references cited by the research collectives (79, 80). The co-citation relationship of documents changed over time, predicting the development and evolution of a discipline through document co-citation networks (81). The numbers of top 10 co-cited references in IL-37 literatures are presented in **Table 5**. In 2015, the most co-cited paper entitled “IL-37 requires the receptors IL-18Rα and IL-1R8 (SIGIRR) to carry out its multifaceted anti-inflammatory program upon innate signal transduction” published by Claudia A. Nold-Petry (35) in *Nature Immunology* demonstrated that the tripartite ligand–receptor complex IL-37–IL-1R8–IL-18Rα scanned by three-dimensional wide-field fluorescence imaging is a prominent form of endogenous IL-37 in the innate immune response. The author clarified that mice with a transgenic expression of IL-37 (IL-37tg) were protected against endotoxemia, whereas IL-1R8-deficient IL-37tg mice were not. Lipopolysaccharide rapidly stimulated the tripartite complex assembled on the surface of peripheral blood mononuclear cells. Therefore, this research not only demonstrated that the extracellular form of IL-37 exerts an immunomodulatory effect but also suggested that it could exert an immunosuppressive role in an inflammatory environment. That same year, Suzhao Li (5) from the University of Colorado Denver further emphasized that IL-1R8 is required for the immunosuppressive effect of extracellular IL-37. Mechanistically, it has been proven that extracellular IL-37 binds to IL-18Rα and recruits IL-1R8 to activate downstream signaling pathways, while the intracellular matured IL-37 binds to Smad3 in the cytoplasm and traffics to the nucleus to regulate gene transcription (14). Although the IL-37/IL-18Rα/IL-1R8 trimeric complex is the extracellular form of immunomodulatory effect of IL-37, it is controversial whether there is another unique receptor for IL-37.

Apart from these significant findings, the mechanisms of IL-37 mediating resistance to innate response have been studied intensively in another publication entitled “IL-37 is a fundamental inhibitor of innate immunity” with 83 co-citations ranking the fourth in the 10 most productive co-cited references published by Marcel F. Nold (2) in 2010. The expression of pro-inflammatory cytokines IL-1α and IL-6 decreased by 88% and 86%, respectively, in RAW cells, and this immunomodulatory role of IL-37 depends on Smad3. Ana-Maria Bulau (4) has proven the role of caspase-1 in the nuclear translocation and function of immunosuppression of IL-37, as well as the process of secretion of mature IL-37. These four studies elaborate on the mechanisms of the intracellular and extracellular immunosuppressive roles of IL-37 in innate response, while having served as a foundation for this new cytokine of IL-37 research in future studies. Since DCs bridge innate and adaptive immunity, DCs from IL-37tg mice exhibited a characterization of immunological tolerance with a lower ability to stimulate syngeneic and allogeneic naive T cells as well as stimulate the increase of Treg (36). In addition to mechanism research, three productive co-cited papers determined the effect of IL-37 in systemic lupus erythematosus, colitis, and rheumatoid arthritis (12, 37, 38). Besides, two reviews of the top 10 co-cited references focused on a summary of the role of IL-37 in innate and acquired immunity (76, 82).

The occurrence of keywords and burst of the network reflect the hotspots of the specific field (83). The high-frequency keywords include “expression”, “IL-37”, “inflammation”, “cytokine”, “IL-1 family”, “cell”, “immunity”, “receptor”, “rheumatoid arthritis”, and “activation”. IL-37, the search term with the frequency of 165, should be excluded from further analysis. Combined with the strongest citation bursts of keywords, the majority of relevant publications during the budding stage from 2000 to 2004 concentrated on the basic structure and mechanism of IL-37. The article entitled “Identification and Initial Characterization of Four Novel Members of the Interleukin-1 Family” published by Sanjay Kumar (43) in 2000 identified the four novel members of the IL-1 family which were designated as IL-1 homologue 1–4 (IL-1H1-4) for the first time and involved in the pathogenesis of immune processes. On the basis of this study, Sanjay Kumar (8) published an article that revealed that caspase-1 cleaves IL–1H4 (renamed IL-1F7b) to attain a mature status. Furthermore, both precursors and mature IL-1F7b could bind to the IL-18Rα receptor of the cell surface. After that, the academics were almost silent on IL-37 research until its reemergence in 2010. Insufficient attention was paid to IL-37 until some scholars at the University of Colorado Denver have conducted in-depth research on the mechanism of IL-37. As showcased in **Figure 1**, the study of IL-37 has been in the rapid development phase since 2010. The keywords with high co-occurrence, “Inflammation”, “Dendritic cell”, “Rheumatoid arthritis”, “Pathogenesis”, and “Disease”, proved that the role of IL-37 in different cellular immune responses and immunological diseases has grown into a main focus of research in the past few years. Emerging evidence revealed that IL-37 suppresses the maturation of DCs and the production of IL-6, IL-1α, and TNF-α in innate immune cells (17). The co-occurrence of keywords predicts that the mechanistic role of IL-37 in a variety of diseases remains the most attractive research subject. The research fronts of IL-37 are still to be confirmed by citation bursts of the keywords. The keyword “IL-1 receptor antagonist (with a burst strength of 2.21)” occurred in 2001 and lasted 13 years, indicating that the research on the inhibitory effect of IL-37 for the IL-1 receptor family persisted over long periods. Likewise, the burst of “receptor (with a burst strength of 2.51)” lasted 11 years. IL-37 weakly binds to IL-18Rα and requires the IL-1 family decoy receptor IL-1R8 constituting the tripartite ligand–receptor complex to exert function (84). Therefore, the studies concerning the IL-18Rα and IL-1R8 lasted for a long time. In addition, the keyword “suppression” with the largest burst strength of 5.81 started to burst from 2019 to the present, which implies that the detection of immune effects of IL-37 in a wide variety of immune disorders still remains a research hotspot in future studies.

The analysis of clusters of co-citations contributed to our understanding of the knowledge structure and dynamic evolution of IL-37 research. Cluster#4 (complex) and cluster#9 (IL-1f5) initiated early, indicating that they are fundamental and matured theoretical knowledge of IL-37 research. The nodes scattered along the timeline of cluster#4 and cluster#9 contain few citations. The main topic involved in the clusters is to identify the role of IL-37 and its receptor from 1996 to 2008. In this period, the article with the most citations published by Philip Bufler (85) discovered that recombinant human IL-37 sharing two conserved amino (Glu-35 and Lys-124) acids with IL-18 was shown to bind to IL-18Rα but failed to recruit the IL-18Rβ chain to form a functionally active complex. Another study in this cluster with the highest betweenness centrality (0.23) analyzed the antitumor activity of human IL-37 by adenovirus-mediated gene transfer (adIL-37) and found that the direct single intra-tumoral injection of adIL-37 resulted in a significant growth suppression of mouse fibrosarcoma at the first attempt (86). Notably, the article with the strongest burst of 3.34 originally identified the initial characterization of four novel members of the IL-1 family, namely, IL-1H1, IL-1H2, IL-1H3, and IL-1H4, which was renamed as IL-37 in 2010 (43). Above all, the integrated cluster with 69 references provides a new vision of IL-37. Cluster #1 (disease activity) has the largest nodes and contains eight of the 10 most frequently cited references from 2010 to 2017. The evolution of this specialty can be divided into two parts. On the one side, the specific mechanism of IL-37 in innate response was further confirmed by transgenic mouse and visualized experiment techniques. On the other hand, a growing body of research has been performed to identify the correlation of IL-37 in several disorders such as mycobacterial infection, colitis, SLE, RA, insulin resistance, and psoriasis (12, 37, 38, 87–89) Similarly, cluster #7 (disease pathogenesis) and cluster #1 (disease activity) still focus on the underlying mechanisms. Cluster #0 (cardiovascular disease) is the largest cluster with 124 references from 2010 to the present. The underlying mechanisms of IL-37 regulating immune responses have been mainly discussed in cluster#1. Therefore, core contents in this cluster still refer to immune-related diseases, especially cardiovascular diseases. The reference with the strongest burst (8.06) demonstrated that IL-37 protects against the development of atherosclerosis in ApoE^-/-^ mice by eliminating the maturation of DCs (90). Similarly, DCs from human atherosclerosis and acute coronary syndrome patients pretreated with rhIL-37 induce tolerance by converting immature DCs into tolerogenic DCs (91). IL-37 has recently been considered to enhance Treg abundance and the production of anti-inflammatory cytokines in patients with atherosclerosis and acute coronary syndrome (1, 92). The levels of IL-10 and TGF-β secreted by T cells isolated from patients were decreased in the treatment of rhIL-37 (91). Although the role of IL-37 in the animal model and clinical patients with atherosclerosis (93–96), acute coronary syndrome (1, 97), coronary artery calcification (98), coronary heart disease (99), chronic heart failure (100), atrial fibrillation (101), abdominal aortic aneurysm (102), hypertension (103), and other cardiovascular system diseases (91) has been identified, the timeline view of co-citation clusters revealed that the relationships between IL-37 and the cardiovascular system diseases remain the future hotspots. Cluster #0 seems to be the transfer of the second part of cluster #1 which has been undertaken in terms of research content. The content from clusters #0 and #1 proved that the specific role of IL-37 in several diseases remains an important direction for future research, consistent with the findings from keyword occurrence and burst. The majority of studies in cluster#0 were all conducted prior to 2010. Therefore, the contents of cluster #2 (differential expression) probed the mechanism of IL-37. Although clusters #2 and #1 partially overlap in content, the reference in cluster#2 has fewer citations. Cluster #5 (related cytokine) is a comprehensive literature collection of the IL-1 family of cytokines. As we know, IL-37 is a newly discovered member of the IL-1 family (104). IL-1α, IL-1β, IL-1Ra, IL-18, IL-33, IL-36α, IL-36β, IL-36γ, L-36Ra, IL-37, and IL-38 have always been summarized in the same article (105, 106). Cluster #6 (mast cell) contains fewer nodes on the timeline, suggesting that the research theme was a previous research hotspot. The research in cluster #8 (epidermal barrier) started in 2013 and continued to the present, implying that the role of IL-37 on the epidermal barrier is a research front and predominant aspect in the following study. An infant with non-functional IL-37 had suffered from inflammatory bowel disease, proving the importance of IL-37 in the function of mucosal immunity and epidermal barrier (107). However, related studies are very scarce and should be further explored. Taken together, we found that the role of IL-37 has received much attention in the past 20 years since its finding. Currently, the broad immune regulatory function of IL-37 is still the focus and continues to be a hotspot in future research.

There are still some limitations in the study. Although some articles referring to IL-37 research included in the WoSCC database may lead to few comprehensive research results, there is no denying that the retrieved literature obtained from the WoSCC database accompanied the features with the reliability of the publications and citations. Furthermore, specific data formats downloaded from the WoSCC database are more convenient and widely accepted for processing in the CiteSpace software. The overwhelming majority of bibliometrics articles have utilized the data from WoSCC. Notably, terms extracted from title, abstract, and keywords exhibit a high discrepancy in the process of clustering analysis due to the certain limitations inherent in CiteSpace software. Besides, some terms with the same meaning should be incorporated using CiteSpace in the co-occurrence of keywords.

## Conclusion

In summary, IL-37 research is undergoing rapid development worldwide, of which USA stands out as the most important contributor to this field. By detecting the most recent burst keywords and co-cited reference clusters up until 2022, emerging trends in IL-37 research were discerned. It is believed that thorough analyses of the immune function of IL-37 in different disorders will be an emerging focus. This bibliometric analysis provides the visualization to the field of IL-37, contributing to tracking the intellectual structure of specific knowledge domains and research fronts of IL-37.

## Author contributions

Y-FQ: conception and design, data analysis and interpretation, manuscript writing. S-HR and BS: collection and assembly of data, data analysis, and interpretation. HQ, HW, G-ML, Y-LZ, C-LS, CL, and J-YZ: collection of data and data analysis. HW: conception and design, financial support, administrative support, manuscript writing, and final approval of the manuscript. All the authors have read and approved the final content of this manuscript.

## Funding

This work was supported by grants to HW from the National Natural Science Foundation of China (No. 82071802), Science and Technology Project of Tianjin Health Commission (No. TJWJ2021MS004), Tianjin Key Medical Discipline (Specialty) Construction Project, and Natural Science Foundation of Tianjin (No. 19JCYBJC27100).

## Conflict of interest

The authors declare that the research was conducted in the absence of any commercial or financial relationships that could be construed as a potential conflict of interest.

## Publisher’s note

All claims expressed in this article are solely those of the authors and do not necessarily represent those of their affiliated organizations, or those of the publisher, the editors and the reviewers. Any product that may be evaluated in this article, or claim that may be made by its manufacturer, is not guaranteed or endorsed by the publisher.
